# Physical Aspects, Phytochemical Profiles, and Nutritional Properties of Lemon (*Citrus limon*) Slices Under Different Drying Technologies

**DOI:** 10.3390/foods14152586

**Published:** 2025-07-23

**Authors:** Zhirong Wang, Qingqing Fu, Guijie Hao, Yuanwei Gu, Tianqi Sun, Lu Gao, Bo Wang, Shuai Wang, Xiangfeng Zheng, Zhenquan Yang, Shengqi Rao

**Affiliations:** 1 School of Food Science and Engineering, Yangzhou University, Yangzhou 225127, China; 15751221063@163.com (Q.F.); yuanweigu0902@163.com (Y.G.); sky0466@yeah.net (T.S.); gaolu@yzu.edu.cn (L.G.); wb@yzu.edu.cn (B.W.); zxf@yzu.edu.cn (X.Z.); yangzq5730@163.com (Z.Y.); 2 Key Laboratory of Healthy Freshwater Aquaculture, Ministry of Agriculture and Rural Affairs, Huzhou Key Laboratory of Aquatic Product Quality Improvement and Processing Technology, Zhejiang Institute of Freshwater Fisheries, Huzhou 313001, China; haoguijie79@126.com; 3 Yangzhou Institute for Food and Drug Control, Yangzhou 225106, China; wshuai608@163.com; 4 Key Laboratory of Catering Food Processing and Safety Control, China General Chamber of Commerce, Yangzhou University, Yangzhou 225127, China

**Keywords:** lemon slice, drying method, phytochemical preservation, bioactive compounds, volatile components, antioxidant activity

## Abstract

Dried lemon slices (LSs) have become increasingly popular as a healthful beverage when infused in hot water. This study examined the effects of freeze drying (FD), hot air drying (HAD), heat pump drying (HPD), and far-infrared drying (FID) on the quality of dried LSs and their brewed beverages. The results show that FD-LSs and their corresponding beverages have the most appealing appearance and maximum levels of ascorbic acid (2.47 and 0.80 mg/g, respectively), synephrine (8.15 and 0.94 mg/g, respectively), and the overwhelming majority of natural and available phenolic compounds, as well as the strongest antioxidant activity, although numerous volatile compounds in FD-LSs were in the lowest abundances. HPD-LSs exhibited similar trends to FD-LSs but contained the peak concentrations of limonene (2258.87 μg/g), γ-terpinene (704.19 μg/g), β-pinene (502.92 μg/g), and α-pinene (188.91 μg/g), which were the four most abundant volatile compounds in dried LSs. Additionally, active ingredients in HPD-LSs generally featured relative high levels of available amounts. In contrast, HAD- and FID-LSs typically displayed unfavorable coloration and low retention levels of natural and available active ingredients. Consequently, FD and HPD demonstrate superior suitability for the commercial-scale production of dried LSs.

## 1. Introduction

The lemon (*Citrus limon*), a hybrid derived from the citron (*C. medica*) and lime (*C. aurantifolia*), is extensively cultivated in the Mediterranean coast, America, and Southeast Asia. It has emerged as one of the most economically significant members of the *Citrus* family, with the main producers being China, India, Italy, the United States, and southern Europe, attributed to its appealing color, pleasant aroma, and nutritional benefits [1,2,3]. Furthermore, the lemon is highly regarded for the presence of numerous bioactive compounds, including ascorbic acid (AsA), organic acids, terpenes, limonoids, phenolic acids, flavonoids, etc., contributing to the recognition among consumers of its considerable medicinal antimicrobial, anti-inflammatory, anticancer, antidiabetic, anti-obesogenic, anti-urolithic, and anti-cardiovascular disease effects [2,4,5]. For instance, the essential oils (terpenes) and phenolic compounds found in lemon exhibit strong antioxidant properties and are utilized as natural sources of functional ingredients, non-toxic preservatives, and active constituents in film matrixes. Notably, lemon peel essential oil has demonstrated substantial inhibition of the activities of α-amylase and α-glucosidase, with IC_50_ values of 8.16 μg/mL and 7.56 μg/mL, respectively [6]. Additionally, phenolic compounds in lemon such as quercetin, hesperidin, and rutin possess skin-whitening and wrinkle-reducing properties, mitigating the damage caused by ultraviolet radiation to human skin [4,7].

As one type of complex biomass with high organic and moisture content, and metabolic activity, however, the lemon is particularly vulnerable to pathogenic fungal attacks and significant losses during distribution, leading to a substantially reduced shelf life and commercial value [8,9]. Moreover, unlike other citrus fruits (e.g., mandarin, orange, pomelo, grapefruit, etc.), the lemon possesses a high acidity level, rendering them unsuitable for direct consumption. Consequently, the lemon is consumed either fresh or predominantly through industrial processing into products such as dried lemon slices (LSs), juices, jams, flavoring tea, condiments, and spices [10]. Recently, dried LSs have gained increasing consumer acceptance as a healthful beverage when infused in hot water, appealing to diverse age demographics [11]. Furthermore, drying, a traditional and effective food preservation method, serves to remove moisture, generate fragile structures, inhibit enzyme activities and biochemical reactions, and prevent microbial growth and reproduction, thereby significantly extending product shelf life, reducing transportation and storage costs, and improving processability [12,13]. Studies have demonstrated that drying can extend the typical shelf life of fresh lemons from one month to ten months without any chemical preservatives [9].

Drying is a multifaceted process characterized by the simultaneous transfer of heat and mass under transient conditions. The drying process is extremely variable, and the quality of the dried product is considerably governed by minor variations of operational parameters, especially temperature. Although elevated drying temperatures coupled with shortened processing durations typically enhance the degradation of thermolabile bioactive compounds, this approach frequently leads to final products with compromised nutritional value and organoleptic properties [3,12,14]. Most commercially available LSs are dried using hot air drying (HAD), resulting in products that not only exhibit shrinkage and browning but also experience a loss of physiologically active ingredients, heat-sensitive nutrients, and associated flavors [3,15]. Various innovative drying techniques, such as heat pump drying (HPD), freeze drying (FD), far-infrared drying (FID), etc., are currently employed for the dehydration of lemons, each offering distinct advantages and disadvantages [3,15,16]. Despite the growing interest in these methods, there is a notable lack of information regarding the effects of different drying methods on the color, aroma, nutrients, and active ingredients of dried LSs and their brewed drinks.

Within this framework, the present study aimed to examine the influence of various drying technologies, including HAD, HPD, FD, and FID, on the physical properties, nutritional composition, and phytochemical profile of LSs, along with their brewed beverages.

## 2. Materials and Methods

### 2.1. Chemicals and Reagents

High-performance liquid chromatography (HPLC)-grade (≥99.9%) methanol, acetonitrile, and formic acid and analytical-grade AsA and NaH_2_PO_4_ were obtained from Macklin Biochemical Co., Ltd. (Shanghai, China). Gas chromatography (GC)-grade (≥98%) C_7_-C_40_ n-alkanes was provided by Push Biotechnology Co., Ltd. (Chengdu, China). Reference standards (≥98%) of synephrine, limonoids, and phenolic compounds are listed in Supplementary File Table S1. Anthrone, 2,6-dichloroindophenol, and NaOH standard solution were obtained from Yuanye Biotechnology Co., Ltd. (Shanghai, China). Trolox, 1,1-diphenyl-2-picrylhydrazyl (DPPH), and 2,2′-azinobis (3-ethylbenzothiazoline-6-sulphonic acid) (ABTS) free radical scavenging ability kits and total antioxidant capacity assay kit were procured from Solarbio Technology Co., Ltd. (Beijing, China).

### 2.2. Fruit and Sample Preparations

Fresh lemon fruits of uniform color and size, devoid of physical damage or insect infestation, were sourced from Longtai Town, Anyue County, Ziyang City, Sichuan Province, China (latitude 30°10′ N, longitude 105°33′ E), renowned as “the hometown of Chinese lemon”. The fruits were thoroughly cleaned, naturally air-dried, randomly grouped, and sliced into approximately 4.0 mm thick sections. A precise quantity of LSs was weighed and dried at 105 °C until a constant weight was achieved to determine the initial moisture content. Meanwhile, the LSs were arranged in a single layer and subjected to four drying processes, FD, HAD, HPD, and FID, in accordance with the previously established protocol with slight modifications [16,17]. The drying procedures were as follows: (a) LSs pre-frozen at −80 °C for 4 h were freeze-dried in a freeze dryer (SCIENTZ-50F, Scientz Biotechnology Co., Ltd., Ningbo, China) with heating plate at 25 °C, cold trap below −50 °C, and vacuum degree less than 10 mbar for 48 h; (b) LSs were dried in an electro-thermal blast oven (BGZ-246, Boxun Industry & Commerce Co., Ltd., Shanghai, China) at (60 ± 1) °C for 20 h with an air velocity at 1.0 m/s; (c) LSs were dried in a heat pump dryer (CN-HGJ12P, Guangzheng Medical Instrument Co. Ltd., Shanghai, China) at (60 ± 1) °C under a relative humidity of 40% for 12 h with a gas of air at 1200 m^3^/h; (d) LSs were dried in a far-infrared drying oven (766-3AS, Huyueming Science Instrument Co., LTD, Shanghai, China) at (60 ± 1) °C for 20 h with a distance between the sample and the infrared heat source of approximately 20 cm. For the latter three hot drying methods, the systems were maintained at the target temperature for 20 min to achieve thermal equilibrium before initiating the drying process. Throughout the hot drying procedures, the weight of the LSs was periodically measured and recorded until the wet basis moisture content fell below 10%. The dried LSs were naturally cooled to room temperature, analyzed for color, and photographed. They were then ground, sieved using a 60-mesh stainless steel sifter, collected, and stored in sealed containers at −80 °C until further analysis.

### 2.3. Analysis of Color Parameters and Browning Degree

The color parameters, including L* (lightness), a* (redness), b* (yellowness), hue angle, chroma, and saturation, for the peels and flesh of LSs were assessed in accordance with the previously established protocol using a colorimeter (LS173, Linshang Technology Co., Ltd., Shenzhen, China) [17,18]. The browning degree was measured using the spectrophotometric method with an ultraviolet–visible spectrophotometer (Evolution One, Thermo Fisher Scientific Instrument Co., Ltd., Shanghai, China), following a procedure analogous to that employed in our earlier research [17].

### 2.4. Analysis of Basic Components

As previously described [19], the total soluble sugar (TSoS) content was determined via anthranone colorimetry using the above-mentioned spectrophotometer; the titratable acid (TA) content was measured using a titration method based on 0.1 mol/L NaOH; determination of AsA content was conducted according to the 2,6-dichloroindophenol titrimetric method.

### 2.5. Analysis of Aroma Compounds

#### 2.5.1. Electronic Nose (E-Nose) Analysis

An E-nose system (cNose, Baosheng Industrial Development Co., Ltd., Shanghai, China), consisting of a sampling device, interaction-sensitive sensor array (Supplementary Table S2), data acquisition system, and data analysis software, was used to analyze the flavor difference of dried LSs. The test conditions were as follows: cleaning time of 120 s and test time of 90 s [17].

#### 2.5.2. GC-Mass Spectrometry (GC-MS) Analysis

The aromatic compositions of LSs were systematically analyzed utilizing headspace solid-phase microextraction (HS-SPME) in conjunction with GC-MS (GP201010200, Shimadzu Corporation, Kyoto, Japan) employing an HP5-MS capillary column (30 m ×  0.25 mm id, 0.25 µm), as per the previous methodology [3,17]. The aromatic constituents of LSs were incubated at 50 °C for 30 min, subsequently adsorbed using a DVB/CAR/PDMS fiber at 50 °C for 40 min, and finally desorbed into the GC-MS for 10  min. The column temperature gradient program commenced at 40 °C for 3 min, followed by a temperature increase at a rate of 17.5 °C/min to 75 °C, 1 °C/min to 80 °C, 2.5 °C/min to 90 °C, and 10 °C/min to 250 °C, with a final hold of 3 min. The injection port temperature was maintained at 250 °C in split mode with a ratio of 1:25, and the detector port was also set at 250 °C. The MS operated in electron ionization mode at 70 eV in the full scan mode. Comprehensive identification of the components was achieved using the NIST 17.0 mass spectrum database, standards of characteristic components, and C_7_-C_40_ n-alkanes. The relative content of each aromatic compound was quantified using decane as an internal standard, presented as μg decane equivalent (DE) per g dry weight (DW). Ultimately, the data matrix was subjected to a principal component analysis (PCA) and partial least squares-discriminant analysis (PLS-DA) to further elucidate sample cluster distributions.

### 2.6. Analysis of Bioactive Compounds

#### 2.6.1. Synephrine Content

Synephrine was extracted with 70% (*v*/*v*) methanol, and its concentration was analyzed using an HPLC system (1260, Agilent, Santa Clara, CA, USA) equipped with an Eclipse Aq-C18 column (250 mm × 4.6 mm, 5 µm) [20,21]. An isocratic elution was performed using 0.06 mol/L NaH_2_PO_4_ (eluent A) and methanol (eluent B) at a flow rate of 0.8 mL/min. The quantification of synephrine was conducted using a diode array detector (DAD) (G1315B, Agilent, USA) at 35 °C with a detection wavelength of 225 nm.

#### 2.6.2. Limonoid Contents

The contents of limonoids, e.g., limonin and nomilin, were determined as previously described [17,22], employing a series of steps including derosination, extraction, centrifugation, drying, reconstitution, collation, and detection via the above-mentioned HPLC-DAD. An isocratic elution of ultrapure water (eluent A) and acetonitrile (eluent B) was applied at a ratio of 6:4. The flow rate was maintained at 1.0 mL/min at a temperature of 30 °C, with detection at a wavelength of 210 nm.

#### 2.6.3. Fraction and Profile Assays of Phenols

Various phenolic fractions, namely free phenols (FPs), esterified-bound phenols (EBPs), glycosylated-bound phenols (GBPs), and insoluble-bound phenols (IBPs), were prepared according to the procedure outlined in Supplementary Figure S1. Subsequently, the above-mentioned HPLC-DAD was used to further quantify the four phenolic fractions at wavelengths of 260, 283, 320, 330, and 367 nm, respectively. Additional methodological details are available in our previous studies [23,24]. Moreover, PLS-DA was performed for the phenolic content matrix to further determine the metabolites ranked by their variable importance in projection (VIP) scores.

### 2.7. Analysis of Active Ingredients and Antioxidant Activities of Lemon Brewed Beverages

An exercise inspired by routine consumption was implemented to examine the profiles and antioxidant activities of accessible active ingredients in LSs. LS powder was precisely weighed and combined with boiled water at 80 °C, maintaining a material-to-liquid ratio of 1:50, and allowed to cool naturally to room temperature. The mixture was then filtered, and the above procedure was repeated twice, with all supernatants being collected. The levels of active ingredients, including AsA, synephrine, limonoids, and phenolic compounds, in the beverages were measured as previously mentioned, and the available amount of each active compound was subsequently shown as a proportion of the total concentration of the respective individual component. Furthermore, the antioxidant capacities of the prepared beverages were assessed through three assays, DPPH and ABTS radical scavenging activity, and ferric reducing antioxidant potential (FRAP), employing commercially available assay kits as per the manufacturers’ protocols. The results are reported as milligrams of Trolox equivalent (TE) or ferrous sulfate equivalent (FSE) per gram of DW [17,25].

### 2.8. Statistical Analysis

All experimental designs were randomized, with each treatment conducted in triplicate. Data are presented as mean ± standard deviation, and a *p*-value of less than 0.05 was considered statistically significant, as determined by Duncan’s multiple range test. OmicShare tools (http://www.omicshare.com/tools) (accessed on 10 January 2025) were utilized to generate PCA, PLS-DA, heatmaps, and bubble plots.

## 3. Results and Discussion

### 3.1. Color Parameters and Browning Degree

Agricultural product color transformation typically decides the acceptability of a product, highlighting its importance as a quality attribute. The Commission Internationale de l’Eclairage (CIELab) color scale is a standardized system for color measurement, in which L* represents lightness (ranging from white to black), a* denotes the green (−) to red (+) axis, and b* represents the blue (−) to yellow (+) axis, with neutral gray at zero for both chromaticity components. Moreover, the values of L*, a*, and b* can be used to calculate hue angle, chroma, and saturation, where hue angle indicates the dominant color tendency, varying continuously from 0 to 360 in the sequence of purplish-red, red, orange-red, orange, yellow, yellow-green, green, and blue-green; while saturation quantifies color intensity and chroma represents the degree of saturation, with higher C and S values indicating a greater color purity and vibrancy, and lower values corresponding to reduced purity and dullness [18,26]. The results related to the appearance, color parameters, and browning degree of LSs are presented in Supplementary File Figure S2 and Table 1. It was evident that FD-LSs effectively preserve the morphology and vibrant yellow color of fresh fruit (Figure S2A), exhibiting peak values for L*, b*, hue angle, and chroma, while showing the lowest values for a*, saturation, and browning degree, irrespective of peel and flesh (Table 1). This finding is closely aligned with the result of Papoutsis et al. [27], who likewise demonstrated that freeze-dried lemon peels maintain the highest hue angle value (reaching up to 92.61). FD is well-regarded for its exceptional capacity to maintain the product quality—encompassing color, shape, and nutritional value, compared to other drying techniques. This is attributed to the absence of heat during the dehydration process and the reduced oxygen conditions, which limit enzymatic activities and degradation reactions [28,29]. Additionally, FD minimizes volume shrinkage and creates a porous structure with a fluffy texture through moisture sublimation [28]. Numerous studies have corroborated that FD is the most suitable drying method for retaining the color and appearance of fruits [17,29], vegetables [14,28], spices [30], and herbs [31]. In contrast, the three heat-drying methods, e.g., HAD, HPD, and FID, exhibited detrimental effects on the color of LSs. This is likely attributable to the elevated temperatures and exposure to open air, which may induce the degradation of compounds sensitive to heat, light, and/or oxygen, as well as initiate biochemical reactions [11,28]. Although the LS peels subjected to these thermal processes did not demonstrate significant differences, statistical analysis revealed substantial effects (*p* < 0.05) on the flesh regarding color parameters and browning degree (Table 1). Notably, LSs processed by HPD and HAD presented a more aesthetically pleasing and acceptable color and appearance than those processed by FID, confirming the trend reported in a prior study [16]. Compared to standard HAD, HPD reduces both drying time and temperature by lowering relative humidity, resulting in a decreased energy consumption and minimized quality loss [32,33]. Consequently, FD and HPD emerge as promising drying techniques for producing dried LSs with superior appearance.

### 3.2. Basic Components

The contents of sugars and acids are pivotal determinants that significantly affect the flavor profile, quality attributes, and longevity of food products. As delineated in Supplementary File Table S3, there were no appreciable effects of drying methods on the contents of TSoS or TA, while treatment significantly affected (*p* < 0.05) the AsA content. This was readily evident in the report, demonstrating a comparable preservation of organic acids and soluble sugars in lemon flavedos regardless of FD or HAD treatment [3]. Notably, FD-LSs showed the highest concentration of AsA at 2.47 mg/g DW, being approximately 2.06-, 1.31-, and 2.17-fold those of HAD-, HPD-, and FID-LSs, respectively. AsA, an efficacious natural antioxidant, must be obtained from the daily diet as humans lack the critical enzyme for its biosynthesis. From this perspective, FD- and HPD-LSs emerge as viable prospects for AsA supplementation.

### 3.3. Aroma Compounds

**Figure 1:**
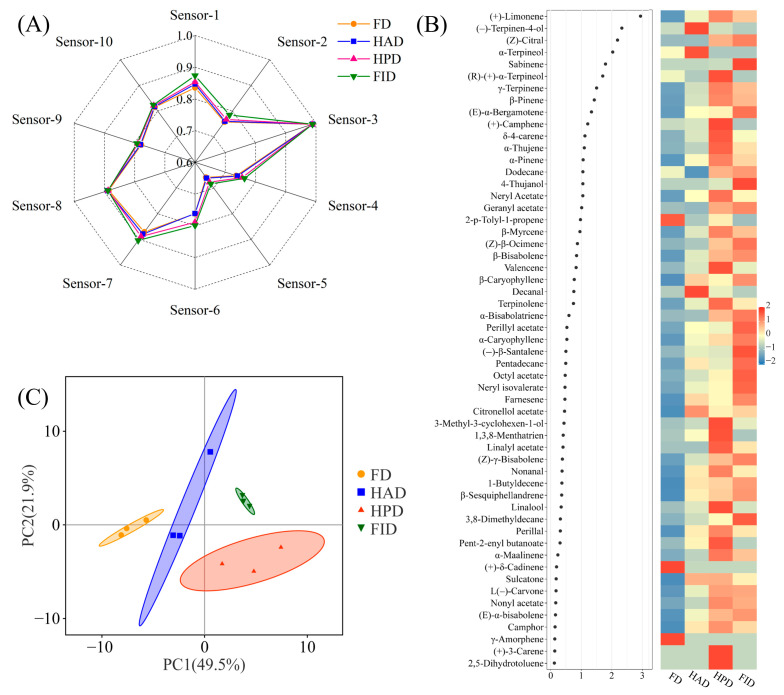
The effects of different drying technologies on aroma similarity (**A**), profiles (**B**), and principal component analysis model (**C**) of volatiles for dried lemon slices. FD, freeze drying; FID, far-infrared drying; HAD, hot air drying; HPD, heat pump drying.

Fruit aroma, dictated by a complex array of volatile organic compounds (VOCs), plays a vital role in flavor quality and significantly contributes to consumer satisfaction. Typically, the aromatic essence of *Citrus* species is predominantly characterized by a diverse spectrum of hydrocarbons, monoterpenoids (C_10_, e.g., limonene, myrcene, phellandrene, pinene, etc.), sesquiterpenes (C_15_, e.g., elemene, caryophyllene, farnesene, etc.), and their oxygenated derivatives [17,34,35,36]. Notably, limonene is the overwhelming predominant constituent in citrus fruits or their essential oils, generally representing 60.44–97.3% of the total VOC content [24,34,35,36]. In this study, the aromatic attributes and VOCs of dried LSs were meticulously assessed using E-nose and HS-SPME-GC-MS techniques, and the results are presented in Figure 1. The E-nose technology is capable of rapidly and accurately assessing the aroma profiles of the volatile-rich samples with a high degree of objectivity, as even minor variations in VOCs can lead to discernible differences in the sensor response values. It can be seen from Figure 1A that markedly stronger responses to the LS-VOCs were detected in sensors 3, 7, and 8. This is in line with previous observations in *C. sinensis* peels [17]. Interestingly, the response values of sensors 3 and 8 showed almost no significant variation across the four types of LSs. However, distinct differences were observed in the response values of sensor 7, with FID-, HPD-, HAD-, and FD-LSs displaying a descending order of response intensity, which implies that FID-LSs contain higher concentrations of inorganic sulfides. Furthermore, FID- and HPD-LSs demonstrated elevated response levels across nearly all ten sensors, particularly in sensors 1, 4, and 6, which are sensitive to benzene aromatic ingredients, hydrides, and methyl-likes, respectively (Figure 1 and Table S2).

A comprehensive qualitative and semi-quantitative analysis of 55 VOCs in LSs was conducted using HS-SPME-GC-MS. The identified VOCs comprised 3 alkanes, 3 alkenes, 6 alcohols, 4 aldehydes, 3 ketones, 13 monoterpene hydrocarbons, 14 sesquiterpenes, and 9 esters. Specifically, 46, 46, 53, and 48 VOCs were detected in FD-, HAD-, HPD-, and FID-LSs, respectively (Figure 1). Precisely, γ-amorphene and (+)-δ-cadinene were exclusively found in FD-LSs, and 2,5-dihydrotoluene and (+)-3-carene were unique to HPD-LSs. Conversely, sulcatone, camphor, and L(−)-carvone were absent solely in FD-LSs; the same traits were observed for dodecane in the HAD samples. Consistent with the E-nose analysis findings, generally, HPD- and particularly FID-LSs exhibited the maximum concentrations of the majority of detectable VOCs. For example, the contents of (Z)-citral, (E)-α-bergamotene, β-bisabolene, and β-caryophyllene in FID-LSs were quantified at 234.96, 130.44, 101.31, and 68.99 μg DE/g DW, being approximately 1.25–43.59-, 1.24–1.81-, 1.05–1.46-, and 1.05–1.29-folds of those in other dried LSs, respectively. In contrast, a great quantity of VOCs, including 3,8-dimethyldecane, 1-butyldecene, linalool, nonanal, α-thujene, α-pinene, terpinolene, farnesene, valencene, nonyl acetate, etc., were present in FD-LSs at their lowest detectable levels. Volatile retention in dried samples is governed by multiple interacting factors, including sample geometry, matrix composition, solid-phase characteristics, initial volatile concentration, and drying rate [3]. The observed reduction in the abundances of VOCs of FD-LSs can be highly attributed to the elevated vacuum degree and prolonged operating time associated with the FD process, evoking the elevated volatilization of VOCs from the plant materials [3,12]. Furthermore, during the initial phase of FD, VOCs exhibit higher vapor pressures compared to ice, leading to their rapid evaporation from both the surface and interior of the frozen material [15]. These results are also supported by previous findings, which demonstrated that FD results in a more remarkable decrease in VOCs in various products, including lemon juice [15], lemon flavedo [3], brocade orange peels [17], andaliman [30], and certain herbs [12].

The obtained data matrices for the relative contents of LS-VOCs were subsequently subjected to PCA and PLS-DA. As depicted in Figure 1C, the PCA scores plot distinctly demonstrated that the first two PCs are sufficient to completely separate all samples, thereby reinforcing the notion that the VOC profiles in LSs are highly governed by the drying methodologies employed. Moreover, within the biplot, the first two PCs succeeded in distinguishing approximately 71.4% of the dried LSs, with PC1 and PC2 accounting for 49.5% and 21.9% of the total variance, respectively. In accordance with established protocols, a VIP score exceeding 1 was utilized as the threshold for variable selection [37]. Consequently, 17 VOCs were identified as providing crucial discriminatory power in the PLS-DA model. These included one alkane, specifically dodecane (VIP score: 1.06); four alcohols, namely (−)-terpinen-4-ol (2.34), α-terpineol (2.04), (+)-α-terpilenol (1.71), and 4-thujanol (1.06); one aldehyde, e.g., (Z)-citral (2.19); eight monoterpene hydrocarbons, including (+)-limonene (2.94), sabinene (1.80), γ-terpinene (1.51), β-pinene (1.43), (+)-camphene (1.22), δ-4-carene (1.13), α-thujene (1.11), and α-pinene (1.07); one sesquiterpene, e.g., (E)-α-bergamotene (1.34); and two esters, namely neryl acetate (1.05) and geranyl acetate (1.02). With the exception of (−)-terpinen-4-ol and α-terpineol, these VOCs were found in their highest concentrations in either HPD- or FID-LSs. Notably, limonene, characterized by citrus, ethereal, and fruity odor (http://www.thegoodscentscompany.com/) (accessed on 10 January 2025), was detected at 2258.87 μg DE/g DW in HPD-LSs, being approximately 2.69-, 1.46-, and 1.17-fold of those in FD-, HAD-, and FID-LSs, respectively. Moreover, γ-terpinene, characterized by its woody, lemon/lime, and herbal flavor; β-pinene, known for its cooling, woody, and piney notes; and α-pinene, with its harsh, aromatic, and minty taste (http://www.odour.org.uk/) (accessed on 10 January 2025), were identified as the second, third, and fourth most abundant VOCs in the dried LSs, partially similar to the results reported by Zhao et al. [3] and Xu et al. [9]. These compounds also reached their highest concentrations in HPD-LSs, attaining 1.13–2.02-, 1.16–3.87-, and 1.31–1.59-folds of those in the other samples, respectively. A notable reduction in the total VOC content has been commonly observed in products subjected to conventional drying methods, including HAD and HPD, considerably attributable to the extensive airflow and extended exposure duration, which facilitate the evaporation and oxidation processes [12]. This trend is especially pronounced for monoterpenes, which, owing to their lower molecular weight, are more readily released from the plant matrix than sesquiterpenes [12,37,38]. The longer duration of thermal drying and the propensity for VOC escape may primarily account for the diminished preservation of VOCs in HAD-LSs relative to the HPD products. Therefore, the results from the E-nose and GC-MS analyses were in substantial agreement, both indicating that HPD- and FID-LSs maintain richer profiles of aromatic compounds.

### 3.4. Bioactive Compounds

#### 3.4.1. Synephrine

**Figure 2:**
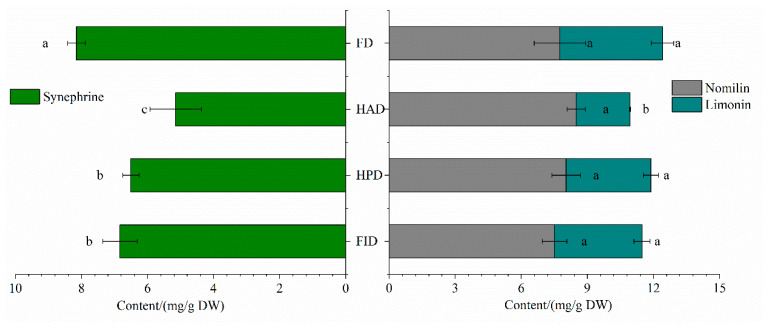
The effects of different drying technologies on contents of synephrine, nomilin, and limonin for dried lemon slices. FD, freeze drying; FID, far-infrared drying; HAD, hot air drying; HPD, heat pump drying. Different superscript letters in a column within the same panel indicate significant differences (*p* < 0.05) by Duncan’s test.

Synephrine is a distinctive and predominant protoalkaloid found in the *Citrus* family. Because of the excellent antihistaminic effect and lipolytic activity, synephrine has garnered increasing interest and has been marketed to consumers as a viable substitute for ephedrine-containing products, which have been prohibited by the Food and Drug Administration [20]. Statistical analysis revealed significant effects (*p* < 0.05) of drying methods on the synephrine contents in LSs. FD-LSs exhibited the highest synephrine concentration at 8.15 mg/g, which was roughly 1.58-, 1.25- and 1.19-fold that of HAD-, HPD-, and FID-LSs, respectively (Figure 2). These findings align well with prior research, which demonstrated that FD preserves synephrine in orange peels better compared to thermal drying methods [17].

#### 3.4.2. Limonoids

Limonin and nomilin are the most prevalent limonoids, characterized by their pronounced bitterness, and responsible for the bitter taste across a majority of *Citrus* species [22,39]. Although bitterness is generally undesirable, limonoids have been attracting increasing attention as they possess a broad spectrum of biological activities and health-promoting benefits [35,40]. Nonetheless, there has been a paucity of data regarding the alterations in their contents throughout the drying process. It is apparent from Figure 2 that, with the exception of a minor discrepancy in limonin content observed in HAD-LSs, no significant variations were detected in the concentrations of nomilin or limonin across the different treated samples. This observation partially lends support to earlier findings by [17], who noted that drying techniques appreciably affect only limonin levels but not the nomilin content in orange peels. A plausible explanation for these minimal differences lies in the fact that nomilin and limonin belong to a category of highly oxidized tetracyclic triterpenoids, which exhibit relatively stable chemical properties.

#### 3.4.3. Phenolic Compounds

In recent decades, phenolic compounds have garnered significant global research interest owing to their potent, multifaceted, and broad-spectrum health-enhancing properties and therapeutic potential [41,42,43]. Phenolic compounds in plant tissues are typically present in various forms, including FPs, EBPs, GBPs, and IBPs [17,42,43]. However, to the best of our knowledge, the influence of drying methods on the compositions and abundances of diverse phenolic fractions have received relatively limited attention.

**Figure 3:**
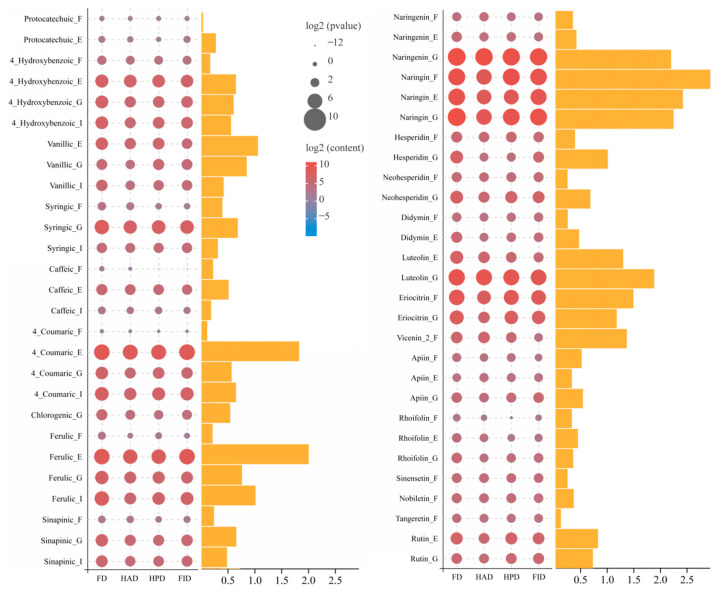
The effects of different drying technologies on profiles and concentrations of phenolic compounds for dried lemon slices. FD, freeze drying; FID, far-infrared drying; HAD, hot air drying; HPD, heat pump drying. In the legend, F, E, G, and I represent free, esterified-, glycosylated-, and insoluble-bound phenols, respectively.

In this investigation, the profiles of phenolic fractions in various dried LSs are presented in Figure 3 and Supplementary Table S4. A total of 23 phenolic compounds were quantified using an HPLC-DAD system. These compounds encompassed four hydroxybenzoic acids, namely protocatechuic, 4-hydroxybenzoic, vanillic, and syringic acids; five hydroxycinnamic acids, including caffeic, 4-coumaric, chlorogenic, ferulic, and sinapic acids; six flavanones, such as hesperidin, neohesperidin, naringenin, naringin, and didymin; eight flavones, namely, luteolin, eriocitrin, vicenin-2, apiin, rhoifolin, sinensetin, tangeretin, and nobiletin; and one flavonol, rutin. The phenolic compositions and concentrations in dried LSs were highly governed not only by drying methodologies, but also by phenolic fractions. EBPs and GBPs typically possessed richer phenolic diversity and markedly higher concentrations (*p* < 0.05) of most components compared to the FPs and IBPs. This result is in good agreement with that of Dong et al. [44], who reported significantly higher concentrations of ferulaic, 4-coumaric acid, vanillic, and 4-hydroxybenzoic acids in EBPs compared to FPs and IBPs in both Eureka lemon peel and pulp tissues.

With minor exceptions, FD-LSs generally exhibited the most diverse phenolic profiles and the highest levels of individual phenolic constituents across various dried samples. Notably, naringin emerged as the predominant compound in the FPs, EBPs, and GBPs. In FD-LSs, the concentration of naringin was significantly higher (*p* < 0.05), reaching 966.43 μg/g DW in FPs, 882.14 μg/g DW in EBPs, and 1538.03 μg/g DW in GBPs, respectively. These values represented increases of 1.02–2.15-, 1.46–2.69-, and 1.65–3.16-folds compared to HAD-, HPD-, and FID-LSs, respectively. Meanwhile, ferulic acid was the most abundant compound in IBPs, with FD-LSs displaying the highest concentration at 216.11 μg/g DW, which was 1.79–3.46-folds that in the other three dried LSs (Figure 3). These findings contrast with the results reported by Gao et al. [45], who identified protocatechuic acid as the predominant phenolic compound in lemons, followed by naringin. This observed variation may potentially result from the differences in lemon cultivars, geographical origins, and extraction methods. Specifically, they did not fractionate the soluble phenolic compounds into FPs, EBPs, and GBPs. In addition to naringin and ferulic acid, FD-LSs revealed the highest concentrations of protocatechuic, 4-hydroxybenzoic, vanillic, syringic, caffeic, 4-coumaric, chlorogenic, and sinapic acids, naringenin, hesperidin, neohesperidin, didymin, luteolin, eriocitrin, vicenin-2, apiin, and rhoifolin, indicating that FD is more effective than other drying methods in preserving phenolic compounds. This observation matches well with considerable prior research that has established FD as a highly effective technique for preserving detectable phenolic acids, flavonoids, and anthocyanins [27,46]. This collective evidence underscored the exceptional ability of FD to preserve bioactive compounds, primarily due to the low-temperature and vacuum conditions employed in FD, which effectively suppress enzymatic activity and decelerate biochemical reactions. Specifically, these conditions mitigate enzymatic browning and the Maillard reaction, thereby reducing the oxidation and degradation phenolic substrates [12,31].

HPD and FID demonstrated comparable effects on phenolic compounds in LSs, with the majority of phenolic components showing no statistically significant differences between HPD- and FID-LSs. While the concentrations of these phenolic compounds were significantly lower (*p* < 0.05) than those observed in FD-LSs, they were still substantially higher (*p* < 0.05) than those in HAD-LSs (Figure 3). This finding aligns with previous studies where HAD samples were generally associated with reduced phenolic contents, indicating that HAD, characterized by prolonged direct air exposure, is less effective in phenolic preservation [28,33]. The inferior performance of HAD may have resulted from oxidative degradation facilitated by extended atmospheric contact during the drying process.

Based on the criterion of VIP scores >1, the effects of drying methods on phenolic compounds in LSs were further evaluated. Thirteen metabolites were identified as the most significant discriminative features, including naringin in FPs, EBPs, and GBPs (VIP score: 2.94, 2.40, and 2.25, respectively); naringenin in GBPs (2.20); ferulic acid in EBPs (1.97); luteolin in GBPs and EBPs (1.89 and 1.31, respectively); 4-coumaric acid in EBPs (1.79); eriocitrin in FPs and GBPs (1.49 and 1.18, respectively); vicenin-2 in FPs (1.37); vanillic acid in EBPs (1.04); and hesperidin in GBPs (1.01) (Figure 3). The distribution of these selected compounds across different fractions revealed three, five, five, and zero metabolites in FPs, EBPs, GBPs, and IBPs, respectively. This pattern suggests that GBPs and EBPs are more susceptible to drying methods compared to FPs, while IBPs remain largely unaffected. This phenomenon may be attributed to the high stability of phenolic complexes in IBPs, where phenolic compounds are tightly bound to macromolecules such as cell wall polysaccharides or proteins [42,43]. According to the principle that a higher VIP value indicates a greater contribution of the metabolite to variable discrimination, naringin emerged as the most significantly altered metabolite in FPs, EBPs, and GBPs. As previously mentioned, naringin also represents the predominant phenolic compound in these fractions. Extensive studies have demonstrated that naringin possesses a wide range of bioactivities [46]. Consequently, FD- and HPD-LSs, which contain elevated levels of naringin, may possess enhanced health-promoting potential.

### 3.5. Active Ingredients and Antioxidant Activities of Lemon Brewed Beverages

**Figure 4:**
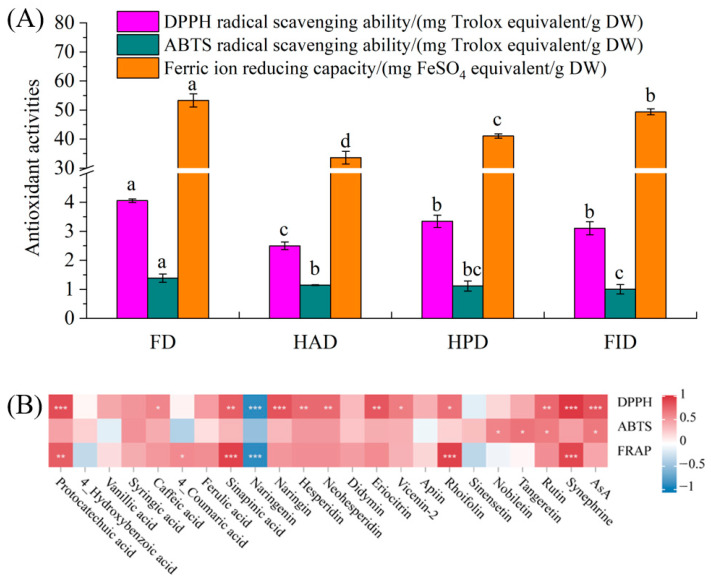
The antioxidant activity (**A**) and the correlation map of antioxidant activity and active ingredients (**B**) of beverages derived from lemon slices under different drying technologies. ABTS, 2,2′-azinobis (3-ethylbenzothiazoline-6-sulphonic acid; AsA, ascorbic acid; DPPH, 1,1-diphenyl-2-picrylhydrazyl; DW, dry weight; FD, freeze drying; FID, far-infrared drying; FRAP, ferric reducing antioxidant potential; HAD, hot air drying; HPD, heat pump drying. Different superscript letters in a column within the same panel indicate significant differences (*p* < 0.05) by Duncan’s test. * Correlation is significant between 0.01 and 0.05, ** correlation is significant between 0.001 and 0.01, and *** correlation is significant at the 0.001 level for Pearson’s correlation coefficient.

To simulate the typical consumption scenario of LSs, the dried LSs were infused in hot water to create beverages, and the available active ingredients and antioxidant activities of beverages were subsequently determined (Table 2, and Figure 4 and Figure S2B). The analysis revealed that chlorogenic acid, luteolin, limonin, and nomilin were not detected. This absence may be caused by the limited water solubility, relatively low abundances, and potential covalent binding to larger biomolecules of these compounds [17,42,43]. In line with the higher concentrations observed in the dried LSs, with the exception of vanillic, and 4-coumaric acids, and naringenin, the beverage derived from FD-LSs contained elevated levels of detectable phenolic compounds, synephrine, and AsA, although a slice of these individual active compounds exhibited a low available amount. Notably, naringin, eriocitrin, ferulic acid, and rutin were the most abundant available compounds, with peak concentrations of 749.04, 119.32, 80.52, and 73.09 μg/g DW in the beverages made by FD-LSs, respectively. These concentrations were approximately 1.06–4.54-, 1.78–1.48-, 1.04–1.12-, and 2.75–3.89-folds those in the beverages prepared by other dried LSs, respectively. However, except for rutin, the highest available amounts of these compounds were observed in the beverages derived from HPD- or HAD-LSs (Table 2). It has been an established fact that, regardless of whether these compounds are sequestered within plant vacuoles or physically/chemically interact with biomacromolecules, their absorption post-consumption is contingent upon their release from the plant matrix [42,43]. As previously noted, the thermal drying process provokes more significant disruption to plant cell structures than that of FD. This disruption further facilitates the release of compounds and allows them to cross cell wall barriers [12,17]. Consequently, even though some phenolic compounds are present at low concentrations in thermally dried LSs, the available amounts of phenols in these samples are generally higher. Among the three thermal drying technologies, available active ingredients of HPD-LSs generally exhibited relatively higher levels of both concentration and amounts, especially vanillic and ferulic acids, naringin, apiin, and AsA (Table 2).

The overproduction of reactive oxygen species (ROS) in the human body can induce oxidative stress, leading to cellular damage and death. Antioxidants mitigate this damage by functioning as part of the body’s antioxidant defense system to neutralize ROS. To evaluate the antioxidant capacity of natural compounds in vitro, assays such as DPPH, ABTS, and FRAP are widely used. These methods are based on the ability of antioxidants to quench stable-colored radicals (DPPH and ABTS) or to reduce metal ions (FRAP), thereby providing a measure of their radical scavenging or reducing capabilities [41]. Correspondingly with the richer abundances of active compounds in FD-LSs, beverages prepared from FD-LSs exhibited significantly higher (*p* < 0.05) DPPH (4.05 mg TE/g DW) and ABTS (1.73 mg TE/g DW) radical scavenging activity, and FRAP (53.30 mg FSE/g DW), compared to other beverages that showed minor differences in antioxidant activity among themselves (Figure 4A). The correlation analysis using the Pearson’s correlation coefficient revealed that DPPH values are significantly positively correlated with (*p* < 0.05) protocatechuic, caffeic, and sinapinic acids, naringin, hesperidin, neohesperidin, eriocitrin, vicenin-2, rhoifolin, rutin, synephrine, and AsA, and extremely significantly negatively related to (*p* < 0.001) naringenin. The ABTS levels showed a significant positive correlation with (*p* < 0.05) nobiletin, tangeretin, rutin, and AsA. FRAP values were significantly positively correlated with (*p* < 0.05) protocatechuic, 4-coumaric, and sinapinic acids, rhoifolin, and synephrine, and extremely significantly negatively correlated with (*p* < 0.001) naringenin (Figure 4B). Partially similar to earlier reports [27,47], these findings are consistent with the notion that the antioxidant activity of phenolic compounds is highly dependent on their structural characteristics, such as the number, nature, and position of substituents on rings A, B, and C, as reported by [41].

### 3.6. Summary of Results

Taken together, the most notable finding from our data is that FD is exceptionally effective in preserving physical coloration (Table 1 and Figure S2A) and nutritional and bioactive compositions (Figure 2 and Figure 3, and Table S3), although it results in a remarkable decrease in the abundances of aroma components (Figure 1). The beverage derived from FD-LSs also exhibited the highest levels of the vast majority of available active ingredients (Table 2) and antioxidant activities (Figure 4). However, it is crucial to acknowledge that FD a fairly time-consuming and costly drying method, primarily due to the operation of vacuum and refrigeration systems, which lead to slow drying rates and higher energy consumption [12,28]. Consequently, the price of commercially available FD-LSs is often several times higher than that of hot-dried LSs. On the other hand, among the tested thermal drying processes, HPD-LSs revealed relatively appealing color (Table 1 and Figure S2A), high levels of both natural and available bioactive compounds (Figure 2 and Figure 3, and Table 2), and aroma compounds (Figure 1), as well as enhanced available amounts of active compounds (Table 2) and antioxidant activities (Figure 4).

## 4. Conclusions

In conclusion, this study systematically elucidated the critical influence of drying technologies on the quality attributes of dried LSs and their corresponding brewed beverages, revealing that FD and, especially, HPD demonstrate attractive performance in preserving physicochemical properties and bioactive constituents. The comprehensive multi-parametric analysis established that these technologies effectively maintain physical aspects, phytochemical profiles, and nutritional properties through their distinctive dehydration mechanisms. Collectively, these findings provide fundamental insights for optimizing industrial processing parameters to enhance product quality. Future research should further identify the specific bioactive markers responsible for the observed health-promoting effects, while parallel efforts need to develop novel stabilization strategies to enhance the bioavailability of these compounds.

## Figures and Tables

**Table 1:** The effects of different drying technologies on color parameters and browning degree for dried lemon slices.

Treatment	Peel						Flesh						Browning Degree
	L*	a*	b*	Hue Angle	Chroma	Saturation	L*	a*	b*	Hue Angle	Chroma	Saturation	
FD	78.91 ± 4.35a	2.09 ± 0.37b	72.26 ± 4.17a	88.35 ± 0.24a	72.30 ± 4.18a	0.92 ± 0.05b	47.30 ± 3.12a	−1.04 ± 0.17d	21.60 ± 2.87a	87.21 ± 0.61d	21.62 ± 2.87b	0.46 ± 0.07c	28.33 ± 2.34d
HAD	54.50 ± 3.72b	16.06 ± 1.22a	55.07 ± 3.86b	73.70 ± 1.29b	57.38 ± 3.84b	1.06 ± 0.08a	24.17 ± 2.42b	4.29 ± 0.56b	25.43 ± 2.66a	80.42 ± 0.61a	25.79 ± 2.70ab	1.08 ± 0.18b	38.04 ± 3.07c
HPD	52.35 ± 4.59b	16.22 ± 2.59a	50.20 ± 5.06b	71.97 ± 3.46b	52.84 ± 4.77b	1.01 ± 0.07ab	12.80 ± 2.05c	12.44 ± 1.80a	23.49 ± 3.29a	61.77 ± 5.27b	26.69 ± 2.86a	2.13 ± 0.15a	48.73 ± 0.81b
FID	52.08 ± 2.15b	13.79 ± 1.83a	54.31 ± 4.67b	75.60 ± 2.74b	56.09 ± 4.29b	1.08 ± 0.08a	0.00 ± 0.00d	−7.73 ± 0.98c	−9.04 ± 1.51b	49.23 ± 4.21c	11.92 ± 1.58c	/	62.06 ± 1.14a

Note: FD, freeze drying; FID, far-infrared drying; HAD, hot air drying; HPD, heat pump drying; /, not calculable. Different letters within a column indicate significant differences (*p* < 0.05) by Duncan’s test.

**Table 2:** The effects of different drying technologies on profiles, concentrations, and available amounts of active ingredients for beverages derived from lemon slices.

Parameter	Available Compound				Available Amount of Compound (%)			
	FD	HAD	HPD	FID	FD	HAD	HPD	FID
Phenols (μg/g DW)								
Protocatechuic acid	2.81 ± 0.22a	1.53 ± 0.13c	1.69 ± 0.12bc	1.82 ± 0.10b	47.75 ± 3.72a	31.79 ± 2.71b	43.84 ± 3.06a	30.10 ± 1.70b
4-Hydroxybenzoic acid	4.98 ± 0.62a	5.12 ± 0.15a	4.98 ± 0.47a	3.69 ± 0.25b	1.52 ± 0.19c	2.69 ± 0.08a	2.29 ± 0.22b	1.71 ± 0.11
Vanillic acid	6.33 ± 0.33b	5.01 ± 0.31c	8.54 ± 0.48a	6.20 ± 0.76b	3.75 ± 0.20b	6.37 ± 0.40a	6.34 ± 0.36a	6.44 ± 0.79a
Syringic acid	37.27 ± 1.54a	29.88 ± 1.29b	25.39 ± 1.80c	25.99 ± 3.22bc	15.15 ± 0.63b	19.67 ± 0.85a	14.25 ± 1.01b	14.67 ± 1.82b
Caffeic acid	10.12 ± 1.64a	6.30 ± 0.02c	7.76 ± 0.30b	6.55 ± 1.51bc	19.95 ± 2.22a	15.85 ± 0.04b	20.70 ± 0.79a	21.76 ± 2.01a
4-Coumaric acid	25.41 ± 0.65b	21.55 ± 1.18c	21.44 ± 0.46c	33.67 ± 3.28a	4.18 ± 0.11c	5.44 ± 0.30a	4.89 ± 0.10b	6.08 ± 0.59a
Ferulic acid	80.52 ± 0.17a	71.97 ± 3.62b	77.50 ± 5.06b	76.80 ± 2.61b	11.60 ± 0.02c	21.47 ± 1.08a	20.20 ± 1.32a	14.60 ± 0.50b
Sinapinic acid	33.65 ± 0.52a	21.30 ± 2.65c	23.47 ± 1.00c	30.01 ± 2.15b	22.34 ± 0.35b	28.46 ± 3.54a	30.89 ± 1.31a	31.72 ± 2.27a
Naringenin	9.89 ± 1.06c	19.64 ± 1.44a	16.46 ± 1.34b	14.09 ± 2.14b	0.68 ± 0.07c	1.58 ± 0.12a	1.43 ± 0.12ab	1.28 ± 0.19b
Naringin	749.04 ± 30.35a	168.16 ± 10.48c	709.30 ± 49.00a	305.23 ± 41.63b	22.12 ± 0.90b	13.31 ± 0.83c	32.64 ± 2.26a	13.12 ± 1.79c
Hesperidin	4.93 ± 0.32a	3.30 ± 0.50b	3.64 ± 0.13b	3.39 ± 0.21b	2.79 ± 0.18c	6.34 ± 0.97a	6.21 ± 0.22a	3.93 ± 0.24b
Neohesperidin	49.30 ± 3.26a	33.05 ± 3.06b	36.44 ± 1.29b	33.87 ± 2.10b	30.91 ± 2.04b	44.20 ± 6.76a	30.96 ± 1.10b	29.38 ± 1.82b
Didymin	21.64 ± 2.85a	16.97 ± 1.98b	13.48 ± 1.79c	18.47 ± 1.78ab	29.74 ± 3.91c	60.56 ± 7.07a	35.90 ± 4.77c	45.19 ± 4.36b
Eriocitrin	119.32 ± 3.26a	80.47 ± 8.41c	101.23 ± 6.98b	83.42 ± 7.07c	16.69 ± 0.46c	29.53 ± 3.09a	18.56 ± 1.28b	17.68 ± 1.50bc
Vicenin-2	8.34 ± 0.91a	6.07 ± 0.66b	6.43 ± 0.81b	5.42 ± 0.22c	11.86 ± 1.29c	8.45 ± 0.92d	15.99 ± 2.01b	43.26 ± 1.79a
Apiin	13.52 ± 1.55ab	11.48 ± 2.02b	14.83 ± 1.30a	13.13 ± 0.74ab	21.22 ± 2.12b	23.02 ± 2.05ab	25.87 ± 2.26a	21.94 ± 1.24b
Rhoifolin	7.58 ± 0.40a	2.83 ± 0.52c	4.14 ± 0.60b	7.12 ± 0.65a	11.91 ± 0.64c	9.49 ± 1.05d	15.94 ± 2.31b	21.12 ± 1.93a
Sinensetin	6.44 ± 0.81ab	6.87 ± 0.49a	5.34 ± 0.92b	4.80 ± 1.05b	24.18 ± 3.06ab	28.24 ± 2.01a	22.30 ± 3.84b	21.72 ± 3.73b
Nobiletin	15.65 ± 0.86a	14.07 ± 1.18a	7.05 ± 0.22b	3.59 ± 0.70c	57.18 ± 3.15a	59.62 ± 4.98a	27.42 ± 0.86b	17.25 ± 1.35c
Tangeretin	13.76 ± 1.27a	9.97 ± 0.89b	8.55 ± 1.31b	2.25 ± 0.14c	55.20 ± 2.32a	47.47 ± 4.22b	40.13 ± 4.44b	10.93 ± 0.66c
Rutin	73.09 ± 8.51a	26.61 ± 3.60b	22.18 ± 4.00bc	18.77 ± 1.98c	54.41 ± 6.33a	38.48 ± 3.21b	17.11 ± 2.08c	14.30 ± 0.51d
Total	1293.61 ± 37.97a	562.16 ± 16.37d	1119.85 ± 63.65b	698.291 ± 56.51c	13.58 ± 0.40b	10.86 ± 0.32c	17.19 ± 0.82a	10.43 ± 0.84c
Other compounds (mg/g DW)								
Synephrine	0.94 ± 0.03a	0.28 ± 0.004c	0.57 ± 0.03b	0.60 ± 0.08b	11.59 ± 0.43a	5.36 ± 0.71c	8.75 ± 0.53b	8.72 ± 0.74b
AsA	0.80 ± 0.07a	0.55 ± 0.04c	0.71 ± 0.04b	0.55 ± 0.07c	32.54 ± 2.77d	45.94 ± 3.24b	37.46 ± 2.08c	65.26 ± 6.37a

Note: DW, dry weight; FD, freeze drying; FID, far-infrared drying; HAD, hot air drying; HPD, heat pump drying. Different letters within a column indicate significant differences (*p* < 0.05) by Duncan’s test.

## Data Availability

The original contributions presented in the study are included in the article, further inquiries can be directed to the corresponding author.

## Supplementary Materials

The following supporting information can be downloaded at: https://www.mdpi.com/article/10.3390/foods14152586/s1, Table S1: The source, retention time, detection wavelength, and calibration curves of tested synephrine, limonoids, and phenol; Table S2: Response features of the sensor array; Table S3: The effects of different drying technologies on contents of basic components for dried lemon slices; Table S4: The effects of different drying technologies on profiles and concentrations of phenolic compounds for dried lemon slices; Figure S1: The flowchart describing the procedure for the extraction of free, esterified-, glycosylated-, and insoluble-bound phenols, the same to our previous work [23]; Figure S2: The visual presentation of dried lemon slices produced by freeze drying (FD), hot air drying (HAD), heat pump drying (HPD), and far-infrared drying (FID) (A), and their corresponding brewed beverages (B), from left to right.
