# Supplementary material for: Physical Aspects, Phytochemical Profiles, and Nutritional Properties of Lemon (Citrus limon) Slices Under Different Drying Technologies
Source: Foods. 2025 Jul 23;14(15):2586. doi: 10.3390/foods14152586 (PMC12346658; doi:10.3390/foods14152586)

foods

Article

Physical aspects, phytochemical profiles, and nutritional properties of lemon (*Citrus limon*) slices under different drying technologies

Zhirong Wang[1, 4 *], Qingqing Fu[1], Guijie Hao[2], Yuanwei Gu[1], Tianqi Sun[1], Lu Gao[1, 4], Bo Wang[1, 4], Shuai Wang[3, 4], Xiangfeng Zheng[1, 4], Zhenquan Yang [1, 4], Shengqi Rao[1, 4 *]

1 School of Food Science and Engineering, Yangzhou University, Yangzhou, 225127, Jiangsu, PR China
2 Key Laboratory of Healthy Freshwater Aquaculture, Ministry of Agriculture and Rural Affairs, Huzhou Key Laboratory of Aquatic Product Quality Improvement and Processing Technology, Zhejiang Institute of Freshwater Fisheries, Huzhou 313001, Zhejiang, China
3 Yangzhou Institute for Food and Drug Control, Yangzhou, 225106, PR China
4 Key Laboratory of Catering Food Processing and Safety Control, China General Chamber of Commerce, Yangzhou University, Yangzhou, Jiangsu, 225127, PR China
***** Correspondence: wangzhirongcn@163.com; sqrao@yzu.edu.cn

Citation: Wang Z.; Fu;Q.; Hao, G.; Gu, Y.;S, T.; G, L.; W, B.; W, S.; Z, X.; Y, Z.; R, S. Physical aspects, phytochemical profiles, and nutritional properties of lemon (*Citrus limon*) slices under different drying technologies. *Foods* **2025**,*06*, x. https://doi.org/10.3390/xxxxx

Academic Editor: Firstname Lastname

Received: date
Accepted: date
Published: date

Publisher's Note: MDPI stays neutral with regard to jurisdictional claims in published maps and institutional affiliations.

Copyright: © 2025 by the authors. Submitted for possible open access publication under the terms and conditions of the Creative Commons Attribution (CC BY) license (https://creativecommons.org/licenses/by/4.0/).

Supplementary File Table S1

The source, retention time, detection wavelength, and calibration curves of tested synephrine, limonoids, and phenols.

Category	Retention time /min	Compound	Detection wavelength /nm	Standard curve	R^2	Linear range /(mg/L)	Source
Alkaloid	3.567	Synephrine	225	Y = 67.105X + 49.322	0.9999	0-100	Aladdin Bio-Chem Technology Co., Ltd (Shanghai, China)
Limonoids	3.562	Nomilin	210	Y = 3.7535X-16.445	0.9980	0-1000	Solarbio Technology Co., Ltd (Beijing, China)
	19.333	Limonin	210	Y = 9.2923X + 36.131	0.9994	0-1000	
Phenolic acids	20.242	Protocatechuic acid	260	Y = 83.962X - 76.659	0.9995	0-250	Solarbio Technology Co., Ltd (Beijing, China)
	28.650	4-Hydroxybenzoic acid	260	Y = 107.56X-82.01	0.9995	0-250	
	34.092	Chlorogenic acid	330	Y = 31.293X - 3.8217	0.9994	0-750	
	34.671	Vanillic acid	260	Y = 77.257X - 44.297	0.9995	0-250	
	35.002	Caffeic acid	320	Y = 155.68X - 89.674	0.9996	0-250	
	37.353	Syringic acid	283	Y = 63.003X - 89.003	0.9992	0-250	
	40.058	4-Coumaric acid	320	Y = 190.91X + 58.939	0.9999	0-200	Herbaceous Source Biotechnology Co., Ltd (Nanjing, Jiangsu, China)
	41.638	Ferulic acid	320	Y = 76.951X - 8.4843	0.9996	0-250	Solarbio Technology Co., Ltd (Beijing, China)
	42.112	Sinapic acid	320	Y = 69.285X - 13.515	0.9996	0-250	
Flavonoids	38.897	Eriocitrin	283	Y = 34.608X + 7.9015	0.9995	0-750	Solarbio Technology Co., Ltd (Beijing, China)
	39.340	Vicenein-2	330	Y = 17.375X - 2.472	0.9998	0-750	Push Biotechnology Co., Ltd. (Chengdu, Sichuan, China)
	43.109	Naringin	283	Y = 33.933X -2.1509	0.9992	0-250	Solarbio Technology Co., Ltd (Beijing, China)
	43.442	Hesperidin	283	Y = 37.222X + 2.5728	0.9991	0-250	
	43.810	Rutin	260	Y = 16.682X - 27.215	0.9992	0-250	
	44.192	Neohesperidin	283	Y = 46.338X - 3.2311	0.9989	0-125	
	45.884	Didymin	283	Y = 30.529X - 38.271	0.9994	0-250	Macklin Biochemical Co., Ltd (Shanghai, China)
	46.766	Rhoifolin	330	Y = 51.684X + 1.8852	0.9997	0-250	Push Biotechnology Co., Ltd. (Chengdu, China)

49.118	Naringenin	283	Y = 64.65X -10.095	0.999	0-125	Solarbio Technology Co., Ltd (Beijing, China)
49.865	Hesperetin	283	Y = 62.318X - 21.08	0.9991	0-125	
51.180	Luteolin	260	Y = 13.218X - 36.033	0.9973	0-100	Aladdin Bio-Chem Technology Co., Ltd (Shanghai, China)
52.112	Sinensetin	330	Y = 39.245X - 94.359	0.998	0-2000	Push Biotechnology Co., Ltd. (Chengdu, Sichuan, China)
53.374	Nobiletin	330	Y = 47.103X + 0.6231	0.9993	0-100	Herbaceous Source Biotechnology Co., Ltd (Nanjing, Jiangsu, China)
54.867	Tangeretin	330	Y = 56.733X - 9.6313	0.9995	0-100	Aladdin Bio-Chem Technology Co., Ltd (Shanghai, China)

Note: the detection wavelength of tested individual compound was its respective maximum ultraviolet absorption wavelength which was determined according to the full-band scan.

The information of some tested compounds was the same to our previous work [17, 21, 23-24].

foods

Supplementary File Table S2

Response features of the sensor array.

Sensor No.	Sensor name	Response of sensor
1	W1C	benzene aromatic ingredient
2	W5S	highly sensitive to oxynitride
3	W3C	sensitive to ammonia aromatic ingredient
4	W6S	selective about hydride
5	W5C	sensitive to methane, propane and aliphatic non-polar molecules
6	W1S	sensitive to methyl-like
7	W1W	sensitive to inorganic sulfide
8	W2S	sensitive to alcohols and aldehydes
9	W2W	sensitive to organic sulfide aromatic ingredient
10	W3S	sensitive to methane and aliphatics

Supplementary File Table S3

The effects of different drying technologies on contents of basic components for dried lemon slices.

Treatment	TSoS/%	TA/%	AsA/(mg/g DW)
FD	12.66±1.13a	19.82±0.28a	2.47±0.04a
HAD	11.17±1.06a	19.89±0.087a	1.20±0.01c
HPD	11.52±1.36a	19.93±0.70a	1.89±0.02b
FID	13.27±1.18a	19.75±0.51a	1.14±0.01d

Note: AsA, ascorbic acid; DW, dry weight; FD, freeze drying; FID, far-infrared drying; HAD, hot air drying; HPD, heat pump drying; TA, titratable acid; TSoS, total soluble sugar. Different superscript letters within a column indicate significant differences ($P < 0.05$) by Duncan's test.

Supplementary File Table S4

The effects of different drying technologies on profiles and concentrations of phenolic compounds for dried lemon slices.

Compounds (μg/g DW)	FD	HAD	HPD	FID
Protocatechuic acid_F	2.05±0.20a	1.76±0.16a	1.81±0.22a	1.82±0.16a
Protocatechuic acid_E	3.84±0.47b	3.06±0.21c	2.04±0.11d	4.20±0.68a
4-Hydroxybenzoic acid_F	14.37±0.96a	11.10±1.22b	11.06±2.20b	11.52±1.51b
4-Hydroxybenzoic acid_E	111.59±3.30a	95.17±4.08b	93.81±8.49b	88.15±7.11b
4-Hydroxybenzoic acid_G	103.30±10.40a	44.78±0.82c	53.92±4.32b	56.35±6.42b
4-Hydroxybenzoic acid_I	97.77±8.78a	39.34±3.38c	59.09±3.25b	60.29±2.21b
Vanillic acid_E	83.85±8.12a	47.72±2.71c	62.05±6.49b	30.68±4.02d
Vanillic acid_G	35.01±2.56b	14.96±2.13c	44.25±2.30a	35.74±5.36b
Vanillic acid_I	49.98±7.05a	16.01±0.96c	28.32±3.67b	29.91±1.22b
Syringic acid_F	9.33±0.90a	6.92±0.79b	3.17±1.15c	3.01±1.11c
Syringic acid_G	209.80±11.57a	124.24±7.01c	150.29±18.06b	146.90±13.56b
Syringic acid_I	26.93±4.58ab	20.73±1.41c	24.78±0.90b	27.22±1.50a
Caffeic acid_F	2.08±0.23a	1.21±0.66b	/	/
Caffeic acid_E	42.25±6.81a	33.72±3.74b	32.36±1.01b	26.04±2.74c
Caffeic acid_I	6.36±1.06a	4.81±0.32b	5.10±0.86ab	4.06±0.46c
4-Coumaric acid_F	1.33±0.01a	1.09±0.17b	0.64±0.13c	0.70±0.21bc
4-Coumaric acid_E	366.90±29.60a	278.13±24.40b	272.22±20.98b	383.27±25.64a
4-Coumaric acid_G	89.91±3.06a	39.29±3.63d	58.54±4.20b	51.64±2.14c
4-Coumaric acid_I	150.33±15.62a	77.61±7.10c	107.08±4.47b	118.54±10.17b
Chlorogenic acid_G	42.10±5.36a	19.41±4.54bc	15.45±4.82c	21.09±1.67b
Ferulic acid_F	6.77±0.24a	2.43±0.28c	3.75±0.36b	2.50±0.54c
Ferulic acid_E	343.69±39.78a	204.60±23.43b	216.16±15.01b	355.92±31.29a
Ferulic acid_G	127.54±5.86a	65.63±2.71c	74.20±4.16b	66.06±5.54bc
Ferulic acid_I	216.11±19.16a	62.47±7.25d	89.51±7.86c	120.73±10.82b
Sinapinic acid_F	5.36±0.68a	5.04±0.32a	3.33±0.67b	3.94±0.49b
Sinapinic acid_G	83.51±3.56a	42.18±6.64c	40.65±7.71c	53.73±3.59b
Sinapinic acid_I	61.77±6.43a	27.62±1.94c	32.01±4.18bc	36.97±2.95b
Naringenin_F	15.86±0.86b	18.54±2.28a	13.41±0.24c	12.14±1.30c
Naringenin_E	34.02±3.78a	16.98±1.68bc	19.18±1.94b	15.88±1.58c
Naringenin_G	1392.04±80.01a	1210.36±86.73b	1115.71±111.01b	1072.97±85.48b
Naringin_F	966.43±13.18a	451.26±53.48c	947.07±49.72a	790.56±73.26b
Naringin_E	882.14±70.96a	327.98±25.78c	352.89±38.91c	604.16±65.78b
Naringin_G	1538.03±128.29a	484.51±69.42c	872.80±89.68b	932.41±101.09b
Hesperidin_F	51.06±6.00a	37.64±3.36b	42.41±7.26ab	37.27±4.27b
Hesperidin_G	125.87±16.95a	14.46±3.35c	16.29±2.78c	48.90±1.43b

Neohesperidin_F	29.68±4.18a	22.27±3.64b	25.24±3.81ab	28.19±2.69a
Neohesperidin_G	129.84±4.94a	52.51±3.78c	92.46±3.30b	87.11±6.07b
Didymin_F	19.07±1.07a	13.59±2.84b	16.84±1.13b	19.03±0.99a
Didymin_E	53.70±6.68a	14.42±3.38c	20.71±2.80b	21.85±2.71b
Luteolin_E	143.71±10.72a	67.14±9.76b	42.20±8.42c	29.24±4.64d
Luteolin_G	729.63±59.78a	600.17±48.76b	586.85±27.89b	506.93±47.74c
Eriocitrin_F	482.12±16.57a	203.07±31.16c	366.49±54.82b	304.23±25.36b
Eriocitrin_G	232.86±25.48a	69.44±10.10d	178.94±4.56b	167.58±4.93c
Vicenin-2_F	70.31±2.19a	71.78±2.51a	40.23±3.37b	12.52±2.12c
Apiin_F	15.73±1.22a	11.31±2.26b	13.69±1.95ab	6.88±1.13c
Apiin_E	11.67±0.69b	16.32±3.49a	14.90±2.10a	13.56±2.55ab
Apiin_G	36.33±4.96a	22.23±4.02b	28.75±3.05b	39.42±5.17a
Rhoifolin_F	6.92±1.58a	3.46±1.45b	1.44±0.25c	3.62±.39b0
Rhoifolin_E	20.48±2.90a	12.24±1.09b	6.58±1.28d	8.97±0.99c
Rhoifolin_G	36.24±0.86a	14.09±2.65c	19.02±2.00b	21.14±1.99b
Sinensetin_F	26.63±0.26a	24.32±1.67b	23.94±1.30b	22.10±2.56b
Nobiletin_F	27.36±0.49a	23.61±2.53bc	25.71±3.24ab	20.82±2.01c
Tangeretin_F	17.07±0.24a	14.71±1.35b	14.91±2.60ab	14.43±0.66b
Rutin_E	93.23±7.71a	43.13±6.47c	84.59±8.11ab	78.06±6.27b
Rutin_G	41.11±7.55b	26.04±4.98c	45.04±5.15ab	53.16±4.04a

Note: /, not detected; DW, dry weight; FD, freeze drying; FID, far-infrared drying; HAD, hot air drying; HPD, heat pump drying. F, E, G, and I represent free, esterified-, glycosylated-, and insoluble-bound phenols, respectively. Different superscript letters within a column indicate significant differences ($P < 0.05$) by Duncan's test.

Supplementary File Fig. S1

The flowchart describing the procedure for the extraction of free, esterified-, glycosylated-, and insoluble-bound phenols, the same to our previous work [23].

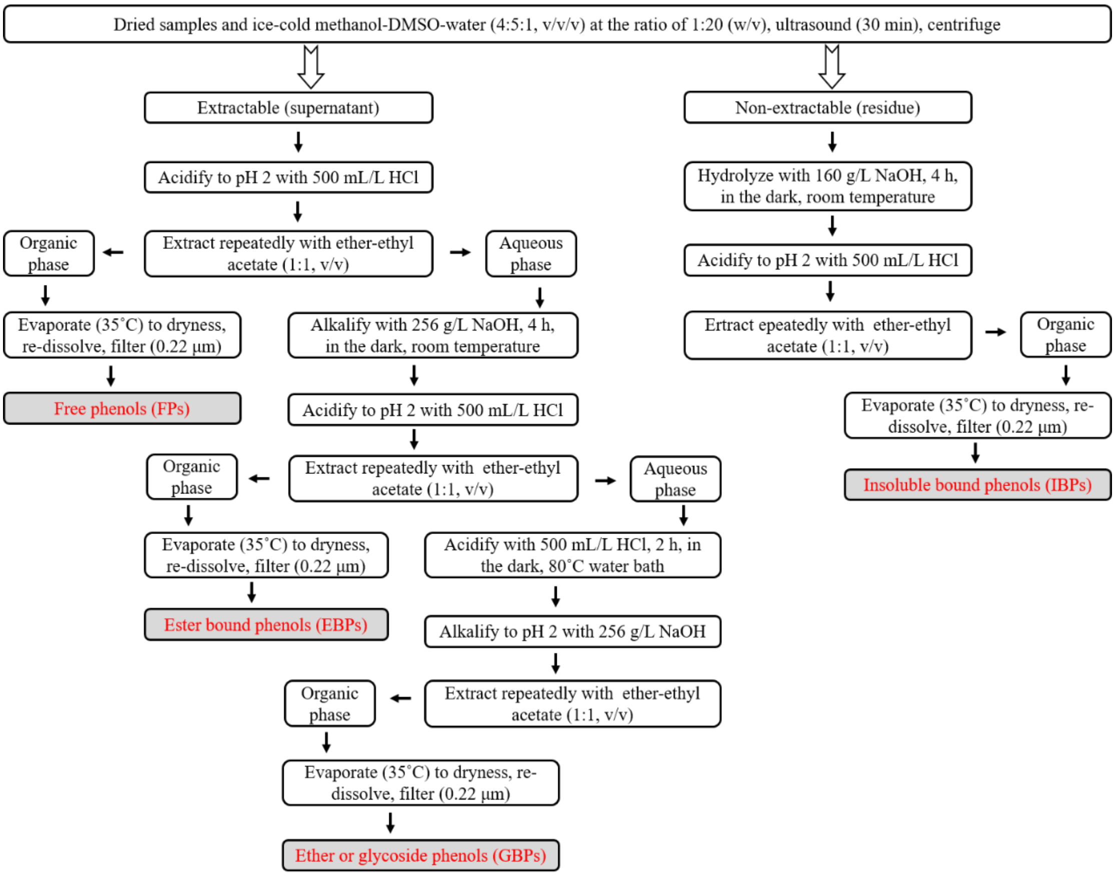

[23] Wang, Z.; Chen, X.; Guo, Z.; Feng, X.; Huang, P.; Du, M.; Zalán, Z.; Kan, J. Distribution and natural variation of free, esterified, glycosylated, and insoluble-bound phenolic compounds in brocade orange (*Citrus sinensis* L. Osbeck) peel. *Food Res. Int.* **2022**, *153*, 110958.

Supplementary File Fig. S2

The visual presentation of dried lemon slices produced by freeze drying (FD), hot air drying (HAD), heat pump drying (HPD), and far-infrared drying (FID) (A), and their corresponding brewed beverages (B), from left to right.

(A)
(B)

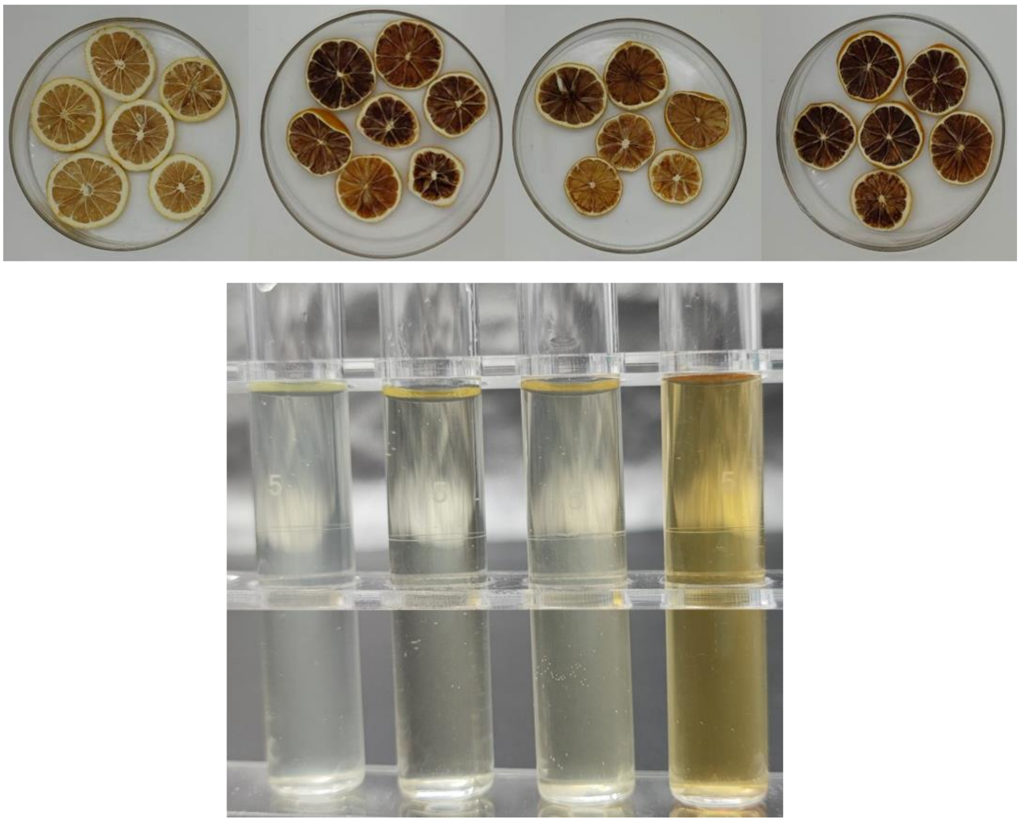

Supplement: Supplementary file 1 [file foods-14-02586-s001.zip › foods-3742325-supplementary.pdf]
